# Antitoxin

**Published:** 1896-08

**Authors:** 


### ANTITOXIN.

The report recently issued to the American Pediatric Society on the antitoxin treatment of diphtheria in private practice is likely to have a distinct effect in increasing confidence in this method of treatment. The total number of cases included in the report was 5,794, of whom 7.3 died, a mortality of 12.3 per cent. But of these 218 were moribund before the treatment was begun, or died within twenty-four hours, and if these be excluded the mortality is reduced to 8.8 per cent. When first used after the third day of the disease the effect of the injections is comparatively slightly visible, and the earlier they are used the better is the result. When employed within the first three days the already mentioned general mortality of 8.8 is reduced to 4.8; yet even when used as late as the fifth or sixth day striking improvement has taken place in some cases.

In three cases the injection had the apparent effect of hastening or causing death. The first, a girl of sixteen, died ten minutes after the injection, in convulsions; in the second, a boy of two and a half years, renal symptom developed, and he died four days later of heart failure; and the third, a boy of three and a half years, died from acute nephritis, after a second injection, but the urine had not been examined before the treatment began.

In only nineteen cases injected reasonably early did the serum appear not to influence the progress of the case, and these were composed of cases in some way out of the ordinary on account of uncertain diagnosis, malignancy of type, etc. Of the 615 physicians from whom returns were received more than 600 spoke favorably of the serum treatment, and generally with enthusiasm. Complications were conspicuous by their comparative absence, the kidneys not appearing to suffer, and the lungs to suffer in much smaller proportion than in hospital cases. The heart appeared to be unfavorably affected on two or three occasions, but its action generally seemed to improve under the treatment.

It was recommended by the committee, which is appointed for another year, that the dose for children under two years of age and for mild cases over that age, should be 1,000 units, repeated if required, and for children over two years, in all laryngeal cases with stenosis, and in all severe cases 1,500 to 2,000 units, to be repeated in from eighteen to twenty-four hours if there be no improvement, a third dose being given if necessary at the expiration of a like time.

